# Psoriasis is characterized by deficient negative immune regulation compared to transient delayed-type hypersensitivity reactions

**DOI:** 10.12688/f1000research.6581.1

**Published:** 2015-06-11

**Authors:** Nicholas Gulati, Mayte Suárez-Fariñas, Joel Correa da Rosa, James G. Krueger

**Affiliations:** 1 Laboratory for Investigative Dermatology, The Rockefeller University, New York, NY, 10065, USA; 2 The Center for Clinical and Translational Science, The Rockefeller University, New York, NY, 10065, USA

**Keywords:** psoriasis, delayed-type hypersensitivity, immune regulation, diphencyprone

## Abstract

Diphencyprone (DPCP) is a hapten that causes delayed-type hypersensitivity (DTH) reactions in human skin, and is used as a topical therapeutic for alopecia areata, warts, and cutaneous melanoma metastases.  We examined peak DTH reactions induced by DPCP (3 days post-challenge) by comprehensive gene expression and histological analysis.  To better understand how these DTH reactions naturally resolve, we compared our DPCP biopsies to those from patients with psoriasis vulgaris, a chronic inflammatory disease that does not resolve.  By both microarray and qRT-PCR, we found that psoriasis lesional skin has significantly lower expression of many negative immune regulators compared to peak DPCP reactions.  These regulators include: interleukin-10, cytotoxic T lymphocyte-associated 4 (CTLA4), programmed cell death 1 (PD1), programmed cell death 1 ligand 1 (PDL1), programmed cell death 1 ligand 2 (PDL2), and indoleamine 2,3-dioxygenase (IDO1).  Their decreased expression was confirmed at the protein level by immunohistochemistry.  To more completely determine the balance of positive vs. negative immune regulators in both DPCP reactions and psoriasis, we developed one comprehensive gene list for positive regulatory (inflammatory) genes, and another for negative regulatory (immunosuppressive) genes, through Gene Ontology terms and literature review.  With this approach, we found that DPCP reactions have a higher ratio of negative to positive regulatory genes (both in terms of quantity and expression levels) than psoriasis lesional skin.  These data suggest that the disease chronicity that distinguishes psoriasis from transient DTH reactions may be related to absence of negative immune regulatory pathways, and induction of these is therefore of therapeutic interest.  Further study of these negative regulatory mechanisms that are present in DPCP reactions, but not in psoriasis, could reveal novel players in the pathogenesis of chronic inflammation.  The DPCP system used here thus provides a tractable model for primary discovery of pathways potentially involved in immune regulation in peripheral tissues.

## Introduction

Diphencyprone (DPCP) is a hapten that induces delayed-type hypersensitivity (DTH) reactions in human skin, and is used therapeutically for alopecia areata ^1^, warts ^2^, and cutaneous melanoma metastases ^3^. The mechanisms by which DPCP decreases pathogenic immunity for the promotion of hair growth in alopecia areata are incompletely understood. DPCP has been shown to alter the cytokine profile in treated alopecic scalp, in particular increasing interleukin (IL)-2 and IL-10 expression ^4^. This increased IL-10 expression has been hypothesized to inhibit the lesional T cells of alopecia areata, but a comprehensive evaluation of other negative immune regulators induced by DPCP is lacking. We have previously shown that human skin responses to DPCP evolve from an inflammatory/effector peak at 3 days post-challenge to a more regulated immune response, with diminished markers of T cell activation, at 14 days. This study included comprehensive gene expression profiling, by microarray and qRT-PCR approaches, of biopsies from DPCP-challenged healthy volunteer skin at 3 days (peak reaction), 14 days (actively resolving reaction), and 120 days (4–8 months; fully resolved reaction) compared to placebo-treated skin ^5^. We have also previously performed similar transcriptomic profiling of psoriasis vulgaris lesional vs. non-lesional skin. This resulted in a meta-analysis derived transcriptome (MAD3) which combined the results of 3 individual microarray experiments, in an effort to address the variability in differentially expressed genes observed between experiments ^6^. In this study, we expand our previous characterizations of transient DTH reactions and chronic psoriasis biopsies by directly comparing them to each other, particularly in relation to positive and negative immune regulation.

## Methods

### Study subjects and skin samples/Consent

For diphencyprone (DPCP) reaction microarray, qRT-PCR, and immunohistochemistry studies, skin biopsies were obtained from 11 volunteers under a protocol approved by The Rockefeller University’s Institutional Review Board (IRB Number JKR-0742). Written, informed consent was obtained from all subjects and the study adhered to the Declaration of Helsinki principles. This trial is registered at clinicaltrials.gov under NCT01452594 ( https://clinicaltrials.gov/ct2/show/NCT01452594). For each volunteer, biopsies were taken of placebo-treated skin as well as DPCP reactions 3, 14, and 120 days after challenge, as previously described ^5^.

For psoriatic lesional vs. non-lesional skin microarray data, we used the meta-analysis derived (MAD3) transcriptome described in 6. Psoriatic lesional tissue for qRT-PCR and immunohistochemistry studies were from deidentified residual samples of plaque-type psoriasis vulgaris from previous studies for whom no clinical characteristics are available; a psoriasis area severity index of more than 12 (moderate-to-severe psoriasis vulgaris with >10% body surface area involvement) was required for entry into these trials.

### RNA extraction, quantification, and microarray

Total RNA was extracted using the miRNeasy Mini Kit (Qiagen, Valencia, CA) according to the manufacturer’s protocol with on-column DNase digestion. The amount of RNA was assessed by NanoDrop 1000 spectrophotometer (Thermo Fisher Scientific Inc., Wilmington, DE). The quality of extracted RNA was examined using Agilent Bioanalyzer 2100 (Agilent Technologies, Palo Alto, CA). RNA was hybridized to HGU133 Plus 2.0 chips (Affymetrix, Santa Clara, CA) to measure relative gene expression.

***Statistical analysis.*** Microarray data were analyzed using R/Bioconductor packages ( http://www.r-project.org). The Harshlight package ^7^ was used to scan Affymetrix chips for spatial artifacts. Expression values were normalized using the GeneChip Robust Multi-array Average (GCRMA) algorithm. Genes with low variation and low expression in most samples were filtered out prior to the analysis. Batch effect due to hybridization date was adjusted using ComBat ^8^. To identify differentially expressed genes, we fitted by REML (Restricted Maximum Likelihood) a linear mixed effect model with treatment (placebo/DPCP) and day (3/14) as fixed effects and a random intercept for each patient. Hypotheses of interest were tested using contrasts in R’s *limma* package framework. The *p*-values resultant from the moderated paired Student’s *t*-tests were adjusted for multiple hypotheses using the Benjamini-Hochberg procedure, which controls for the false discovery rate. The DPCP data discussed in this publication have been deposited in the National Center for Biotechnology Information’s Gene Expression Omnibus (GSE accession number GSE52360, http://www.ncbi.nlm.nih.gov/geo/). Psoriasis data were derived from ^6^.

### Quantitative RT-PCR

Pre-amplification quantitative RT-PCR technique was used for measuring various genes in total RNA extracted from skin biopsy samples according to the company’s instructions. Briefly, 5 ng of total RNA was subjected to first-strand cDNA synthesis using High Capacity cDNA Reverse Transcription kits (Applied Biosystems, Carlsbad, CA). The resulting cDNA was subjected to 14 cycles of pre-amplification using TaqMan PreAmp Master Mix Kit (Applied Biosystems) with desired pooled assay mix. The Gene Amp PCR System 9700 (Applied Biosystems) was used for the pre-amplification reaction with the following thermal cycler conditions: 10 min at 95°C and 14 cycles of 15 seconds at 95°C followed by 4 min at 60°C. 12.5 μl of pre-amplified cDNA was then used for quantitative RT-PCR reaction using TaqMan Gene Expression Master Mix (Applied Biosystems). The 7900HT Fast Real-Time PCR System was used for PCR reactions, and the thermal cycler conditions were as follows: 2 minutes at 50°C, 5 minutes at 95°C, and 40 cycles of 15 seconds at 95°C followed by 60 seconds at 60°C. Data were analyzed by the Applied Biosystems PRISM 7700 software (Sequence Detection Systems, ver. 1.7) and normalized to human acidic ribosomal protein (hARP) housekeeping gene.

All assays were from Applied Biosystems and inventoried assays used in this study were as follows: IL10 (Hs00961622_m1), CTLA4 (Hs03044418_m1), PDCD1 (PD1) (Hs01550088_m1), CD274 (PDL1) (Hs01125301_m1), PDCD1LG2 (PDL2) (Hs01057777_m1), IDO1 (Hs00984148_m1), and LAG3 (Hs00158563_m1). For RPLP0/hARP, a custom primer/probe set was used (Forward: CGCTGCTGAACATGCTCAA, Reverse: TGTCGAACACCTGCTGGATG, Probe: 6-FAM-TCCCCCTTCTCCTTTGGGCTGG-TAMRA).

### Immunohistochemistry

Frozen sections of skin biopsies were dried at room temperature and then fixed for 2 minutes in acetone. Next, the samples were blocked with 10% normal serum of the species in which the secondary antibody was made and then the samples were incubated overnight at 4°C with the appropriate primary antibody. Biotin-labeled secondary antibodies (Vector Laboratories, Burlingame, CA) were amplified with avidin-biotin complex (Vector Laboratories) and developed with chromogen 3-amino-9-ethylcarbazole (Sigma Aldrich, St. Louis, MO) to produce a red color indicative of positive staining.

Primary antibodies used in this study are as follows (all are mouse monoclonal): IL10 (Life Technologies, Clone 945A2A5, IgG1, 1:50 dilution), CD95 (FAS) (BD Biosciences, Clone DX2, IgG1, 1:50), LAG3 (Enzo Life Sciences, Clone 17B4, IgG1, 1:50), PD1 (eBioscience, Clone MIH4, IgG1, 1:50), PDL1 (eBioscience, Clone MIH1, IgG1, 1:50), PDL2 (eBioscience, Clone MIH18, IgG1, 1:100), IDO1 (LifeSpan Biosciences, Inc., Clone 10.1, IgG3, 1:100), and CTLA4 (abcam, Clone BNI3, IgG2a, 1:100).

## Results

**Figure 1.**
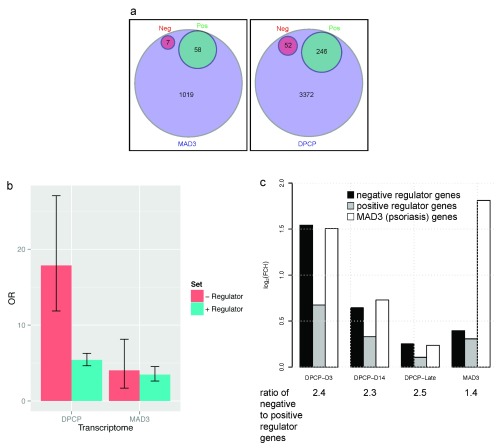
Psoriasis lesional skin has an altered global balance of positive vs. negative regulatory gene transcripts compared to DPCP reactions. ( **a**) Venn diagrams showing overlap of MAD3 psoriasis transcriptome (left) and DPCP day 3 transcriptome (right) with both positive regulatory (Pos) and negative regulatory (Neg) gene lists (common gene lists applied to both transcriptomes). The percentages of the MAD3 and DPCP day 3 transcriptomes comprised of the positive regulatory gene list are 5.7% and 7.3%, respectively. On the other hand, the percentages comprised of the negative regulatory gene list are 0.7% and 1.5%, respectively. ( **b**) Odds ratios (OR) of negative regulatory (red bars) and positive regulatory (blue bars) gene lists in DPCP day 3 and psoriasis transcriptomes. ( **c**) Black bars represent negative regulator genes, gray bars represent positive regulator genes, and white bars represent all MAD3 psoriasis transcriptome genes. The y-axis shows log _2_ (fold change) of all genes in the given gene set. DPCP day 3 and MAD3 samples have comparable MAD3 transcriptome expression levels but there is a substantial difference between all DPCP time points (days 3, 14, and 120 or “late”) and MAD3 samples in terms of the relative levels of negative and positive regulator gene list expression. This is quantified as the “ratio of negative to positive regulator genes.”

Since DTH reactions naturally resolve, we sought to compare our DPCP biopsies (from 5) to those taken from patients with psoriasis vulgaris (from 6), a chronic T cell-mediated inflammatory disease that does not resolve and which, in many ways, represents amplifications of background immune circuits that exist in normal human skin ^9^. To globally assess the balance of positive vs. negative immune regulators in both DPCP reactions and psoriasis using our microarray data, we developed one comprehensive gene list for positive regulatory or inflammatory genes and another gene list for negative regulatory or immunosuppressive genes (through Gene Ontology terms and literature review previously discussed in 5, Table 1 has “negative regulator” list and fold change values for DPCP day 3 and psoriasis transcriptomes, “positive regulator” list is derived from GO term 0002684 “positive regulation of immune system process” but with genes removed that are in common with GO term 0002683 “negative regulation of immune system process”). Our microarray data showed increased fold changes of many negative regulators in DPCP day 3 biopsies vs placebo-treated skin (DPCP day 3 transcriptome) compared to psoriasis lesional vs non-lesional skin (psoriasis transcriptome). For instance, CTLA4 expression was significantly increased 21.6-fold in the DPCP day 3 transcriptome, but non-significantly increased 3.7-fold in the psoriasis transcriptome. Venn diagrams show that the psoriasis transcriptome only has seven genes from the negative regulator list, while the DPCP day 3 transcriptome has 52 ( Figure 1a). Although the DPCP day 3 transcriptome also has more genes from the positive regulator list than psoriasis, the odds ratio for the positive regulator list was not significantly different between these two transcriptomes. The odds ratio for the negative regulator list, however, was significantly different ( Figure 1b). The altered balance between positive vs. negative regulatory transcripts in psoriasis compared to DPCP reactions can also be seen in Figure 1c which shows that DPCP transcriptomes at all time points (days 3, 14, and 120) have a higher ratio of negative to positive regulator genes than the psoriasis transcriptome in terms of expression levels for each gene set as a whole (as opposed to number of genes as indicated in the Venn diagrams). This is despite the fact that the DPCP day 3 transcriptome has comparable expression levels of the MAD3 psoriasis transcriptome genes to actual psoriasis samples, and therefore highlights the negative regulator expression that is unique to DPCP reactions.

**Table 1.** Expression of negative regulator genes in DPCP day 3 vs. placebo and psoriasis lesional vs. non-lesional skin samples.

			DPCP day 3				psoriasis		
Probe	Symbol	Description	FCH	p	FDR		FCH	p	FDR
207526_s_at	IL1RL1	interleukin 1 receptor-like 1	42.8	5.3E-11	2.1E-09		1.1	3.6E-03	1.3E-02
227458_at	CD274	CD274 molecule	34.6	1.9E-12	1.3E-10		23.7	0.0E+00	0.0E+00
236341_at	CTLA4	cytotoxic T-lymphocyte-associated protein 4	21.6	3.7E-11	1.5E-09		3.7	6.0E-02	1.4E-01
207238_s_at	PTPRC	protein tyrosine phosphatase, receptor type, C	18.0	1.6E-12	1.2E-10		2.6	0.0E+00	0.0E+00
206341_at	IL2RA	interleukin 2 receptor, alpha	17.9	4.7E-14	6.3E-12		1.3	1.6E-01	2.9E-01
210146_x_at	LILRB2	leukocyte immunoglobulin-like receptor, subfamily B (with TM and ITIM domains), member 2	12.5	3.6E-08	5.4E-07		2.9	0.0E+00	0.0E+00
222062_at	IL27RA	interleukin 27 receptor, alpha	12.3	9.9E-13	7.6E-11		1.3	1.7E-03	6.7E-03
217192_s_at	PRDM1	PR domain containing 1, with ZNF domain	11.1	8.7E-12	4.6E-10		3.0	0.0E+00	0.0E+00
215719_x_at	FAS	Fas (TNF receptor superfamily, member 6)	9.3	7.8E-07	7.6E-06		1.1	2.8E-02	7.6E-02
205926_at	IL27RA	interleukin 27 receptor, alpha	8.3	5.9E-11	2.3E-09		1.2	1.3E-01	2.4E-01
204780_s_at	FAS	Fas (TNF receptor superfamily, member 6)	8.0	2.6E-05	1.6E-04		1.1	1.3E-01	2.4E-01
242743_at	IL4R	interleukin 4 receptor	8.0	7.6E-13	6.2E-11		1.1	6.0E-02	1.4E-01
242809_at	IL1RL1	interleukin 1 receptor-like 1	6.6	1.5E-06	1.3E-05		1.0	7.6E-01	8.3E-01
207697_x_at	LILRB2	leukocyte immunoglobulin-like receptor, subfamily B (with TM and ITIM domains), member 2	6.6	1.2E-13	1.4E-11		1.5	0.0E+00	0.0E+00
212588_at	PTPRC	protein tyrosine phosphatase, receptor type, C	6.5	2.8E-08	4.3E-07		2.4	0.0E+00	0.0E+00
216252_x_at	FAS	Fas (TNF receptor superfamily, member 6)	6.3	9.4E-08	1.2E-06		1.1	7.0E-04	2.9E-03
230052_s_at	NFKBID	nuclear factor of kappa light polypeptide gene enhancer in B-cells inhibitor, delta	6.2	1.0E-07	1.3E-06		1.4	1.4E-02	4.3E-02
211336_x_at	LILRB1	leukocyte immunoglobulin-like receptor, subfamily B (with TM and ITIM domains), member 1	5.2	4.8E-16	1.5E-13		1.3	2.3E-03	8.7E-03
212587_s_at	PTPRC	protein tyrosine phosphatase, receptor type, C	5.1	6.5E-13	5.5E-11		1.8	2.2E-03	8.3E-03
207104_x_at	LILRB1	leukocyte immunoglobulin-like receptor, subfamily B (with TM and ITIM domains), member 1	5.1	3.0E-17	1.6E-14		1.2	0.0E+00	0.0E+00
211269_s_at	IL2RA	interleukin 2 receptor, alpha	5.1	2.8E-08	4.4E-07		1.1	1.8E-01	3.1E-01
204781_s_at	FAS	Fas (TNF receptor superfamily, member 6)	4.7	3.4E-06	2.7E-05		1.2	3.6E-02	9.3E-02
1552480_s_at	PTPRC	protein tyrosine phosphatase, receptor type, C	4.5	9.0E-08	1.2E-06		1.0	3.3E-01	4.7E-01
203233_at	IL4R	interleukin 4 receptor	4.4	1.2E-10	4.0E-09		3.9	0.0E+00	0.0E+00
206060_s_at	PTPN22	protein tyrosine phosphatase, non- receptor type 22 (lymphoid)	4.3	7.6E-06	5.5E-05		2.3	1.0E-04	4.0E-04
223834_at	CD274	CD274 molecule	3.8	5.7E-09	1.1E-07		1.5	3.6E-03	1.3E-02
235458_at	HAVCR2	hepatitis A virus cellular receptor 2	3.8	6.6E-07	6.6E-06		1.5	1.4E-03	5.5E-03
231794_at	CTLA4	cytotoxic T-lymphocyte-associated protein 4	3.4	1.6E-09	3.7E-08		1.2	6.4E-02	1.4E-01
227900_at	CBLB	Cbl proto-oncogene, E3 ubiquitin protein ligase B	3.3	1.8E-04	8.3E-04		0.8	9.6E-03	3.1E-02
220418_at	UBASH3A	ubiquitin associated and SH3 domain containing A	3.3	3.0E-05	1.8E-04		1.1	2.7E-02	7.3E-02
202643_s_at	TNFAIP3	tumor necrosis factor, alpha-induced protein 3	3.2	1.0E-08	1.8E-07		1.2	1.9E-01	3.2E-01
240070_at	TIGIT	T cell immunoreceptor with Ig and ITIM domains	3.0	2.9E-05	1.8E-04		1.3	4.2E-02	1.0E-01
201537_s_at	DUSP3	dual specificity phosphatase 3	3.0	4.8E-11	1.9E-09		1.5	0.0E+00	0.0E+00
228964_at	PRDM1	PR domain containing 1, with ZNF domain	2.9	3.4E-06	2.7E-05		3.0	0.0E+00	0.0E+00
201538_s_at	DUSP3	dual specificity phosphatase 3	2.9	3.6E-10	1.1E-08		1.3	2.5E-01	3.8E-01
203236_s_at	LGALS9	lectin, galactoside-binding, soluble, 9	2.7	3.5E-07	3.8E-06		1.6	0.0E+00	0.0E+00
224399_at	PDCD1LG2	programmed cell death 1 ligand 2	2.7	1.3E-07	1.7E-06		1.0	1.6E-01	2.8E-01
241889_at	NFKBID	nuclear factor of kappa light polypeptide gene enhancer in B-cells inhibitor, delta	2.6	6.5E-06	4.8E-05		1.0	8.8E-02	1.9E-01
223506_at	ZC3H8	zinc finger CCCH-type containing 8	2.6	5.6E-04	2.2E-03		1.6	0.0E+00	0.0E+00
209744_x_at	ITCH	itchy E3 ubiquitin protein ligase	2.6	3.8E-07	4.1E-06		2.8	1.4E-02	4.1E-02
225622_at	PAG1	phosphoprotein associated with glycosphingolipid microdomains 1	2.6	3.4E-05	2.0E-04		1.9	0.0E+00	0.0E+00
217094_s_at	ITCH	itchy E3 ubiquitin protein ligase	2.4	1.1E-05	7.8E-05		2.4	6.9E-02	1.5E-01
224211_at	FOXP3	forkhead box P3	2.4	1.2E-05	7.9E-05		1.0	0.0E+00	1.0E-04
243196_s_at	TRAFD1	TRAF-type zinc finger domain containing 1	2.3	4.0E-05	2.3E-04		0.9	4.8E-01	6.1E-01
228996_at	RC3H1	ring finger and CCCH-type domains 1	2.3	5.5E-05	3.0E-04		1.8	2.5E-01	3.9E-01
219364_at	DHX58	DEXH (Asp-Glu-X-His) box polypeptide 58	2.3	5.3E-07	5.4E-06		1.3	0.0E+00	0.0E+00
202763_at	CASP3	caspase 3, apoptosis-related cysteine peptidase	2.2	1.8E-05	1.1E-04		1.1	1.1E-01	2.2E-01
236539_at	PTPN22	protein tyrosine phosphatase, non-receptor type 22 (lymphoid)	2.2	6.9E-05	3.7E-04		2.0	0.0E+00	0.0E+00
202644_s_at	TNFAIP3	tumor necrosis factor, alpha-induced protein 3	2.2	1.4E-06	1.3E-05		1.1	2.8E-01	4.2E-01
205298_s_at	BTN2A2	butyrophilin, subfamily 2, member A2	2.2	3.7E-04	1.6E-03		1.1	1.9E-01	3.2E-01
217513_at	MILR1	mast cell immunoglobulin-like receptor 1	2.1	6.0E-06	4.4E-05		1.2	5.9E-02	1.4E-01
205299_s_at	BTN2A2	butyrophilin, subfamily 2, member A2	2.0	3.9E-05	2.2E-04		1.1	1.1E-01	2.2E-01
242497_at	TRAFD1	TRAF-type zinc finger domain containing 1	1.8	4.1E-04	1.7E-03		1.0	6.3E-01	7.4E-01
234066_at	IL1RL1	interleukin 1 receptor-like 1	1.8	7.5E-03	2.0E-02		1.0	3.6E-01	5.1E-01
235668_at	PRDM1	PR domain containing 1, with ZNF domain	1.8	3.0E-06	2.4E-05		2.2	0.0E+00	0.0E+00
1555628_a_at	HAVCR2	hepatitis A virus cellular receptor 2	1.8	6.7E-04	2.6E-03		1.0	4.3E-01	5.6E-01
209682_at	CBLB	Cbl proto-oncogene, E3 ubiquitin protein ligase B	1.8	6.1E-05	3.3E-04		0.8	0.0E+00	0.0E+00
209354_at	TNFRSF14	tumor necrosis factor receptor superfamily, member 14	1.8	3.2E-06	2.6E-05		1.0	7.1E-01	8.0E-01
227354_at	PAG1	phosphoprotein associated with glycosphingolipid microdomains 1	1.7	7.0E-02	1.3E-01		1.3	3.7E-02	9.4E-02
209743_s_at	ITCH	itchy E3 ubiquitin protein ligase	1.7	4.4E-03	1.3E-02		1.2	0.0E+00	0.0E+00
35254_at	TRAFD1	TRAF-type zinc finger domain containing 1	1.7	1.7E-05	1.1E-04		0.9	2.8E-01	4.1E-01
1555629_at	HAVCR2	hepatitis A virus cellular receptor 2	1.7	2.4E-04	1.1E-03		1.0	7.6E-01	8.4E-01
202837_at	TRAFD1	TRAF-type zinc finger domain containing 1	1.6	5.5E-05	3.0E-04		1.0	9.1E-01	9.4E-01
235057_at	ITCH	itchy E3 ubiquitin protein ligase	1.6	2.7E-02	6.0E-02		0.9	2.0E-01	3.3E-01
1554285_at	HAVCR2	hepatitis A virus cellular receptor 2	1.6	5.4E-04	2.2E-03		1.0	1.5E-01	2.7E-01
225626_at	PAG1	phosphoprotein associated with glycosphingolipid microdomains 1	1.5	2.3E-02	5.2E-02		1.3	6.0E-04	2.5E-03
201536_at	DUSP3	dual specificity phosphatase 3	1.5	3.2E-04	1.4E-03		0.8	0.0E+00	1.0E-04
1553042_a_at	NFKBID	nuclear factor of kappa light polypeptide gene enhancer in B-cells inhibitor, delta	1.5	3.0E-03	9.4E-03		1.1	2.6E-02	7.1E-02
220049_s_at	PDCD1LG2	programmed cell death 1 ligand 2	1.4	5.4E-03	1.5E-02		1.0	4.3E-01	5.7E-01
221331_x_at	CTLA4	cytotoxic T-lymphocyte-associated protein 4	1.3	4.8E-04	2.0E-03		1.1	4.7E-02	1.1E-01
234362_s_at	CTLA4	cytotoxic T-lymphocyte-associated protein 4	1.3	4.6E-03	1.3E-02		1.1	1.2E-01	2.4E-01
208010_s_at	PTPN22	protein tyrosine phosphatase, non-receptor type 22 (lymphoid)	1.3	2.4E-03	7.8E-03		1.1	4.0E-04	1.7E-03
234895_at	CTLA4	cytotoxic T-lymphocyte-associated protein 4	1.1	4.9E-02	9.7E-02		1.0	2.0E-04	8.0E-04
225893_at	RC3H1	ring finger and CCCH-type domains 1	1.1	7.0E-01	7.8E-01		1.0	3.6E-01	5.0E-01
224859_at	CD276	CD276 molecule	0.9	4.4E-01	5.6E-01		0.9	2.0E-04	9.0E-04
236235_at	ITCH	itchy E3 ubiquitin protein ligase	0.8	2.6E-01	3.7E-01		0.7	3.6E-03	1.3E-02
239101_at	ITCH	itchy E3 ubiquitin protein ligase	0.7	1.5E-02	3.6E-02		0.7	0.0E+00	0.0E+00
1559583_at	CD276	CD276 molecule	0.6	8.5E-02	1.5E-01		1.0	3.2E-01	4.6E-01
219768_at	VTCN1	V-set domain containing T cell activation inhibitor 1	0.3	8.1E-05	4.2E-04		0.6	2.2E-03	8.6E-03
204472_at	GEM	GTP binding protein overexpressed in skeletal muscle	0.3	1.1E-05	7.6E-05		1.0	3.0E-01	4.4E-01

FCH, fold change; FDR, false discovery rate.

**Figure 2.**
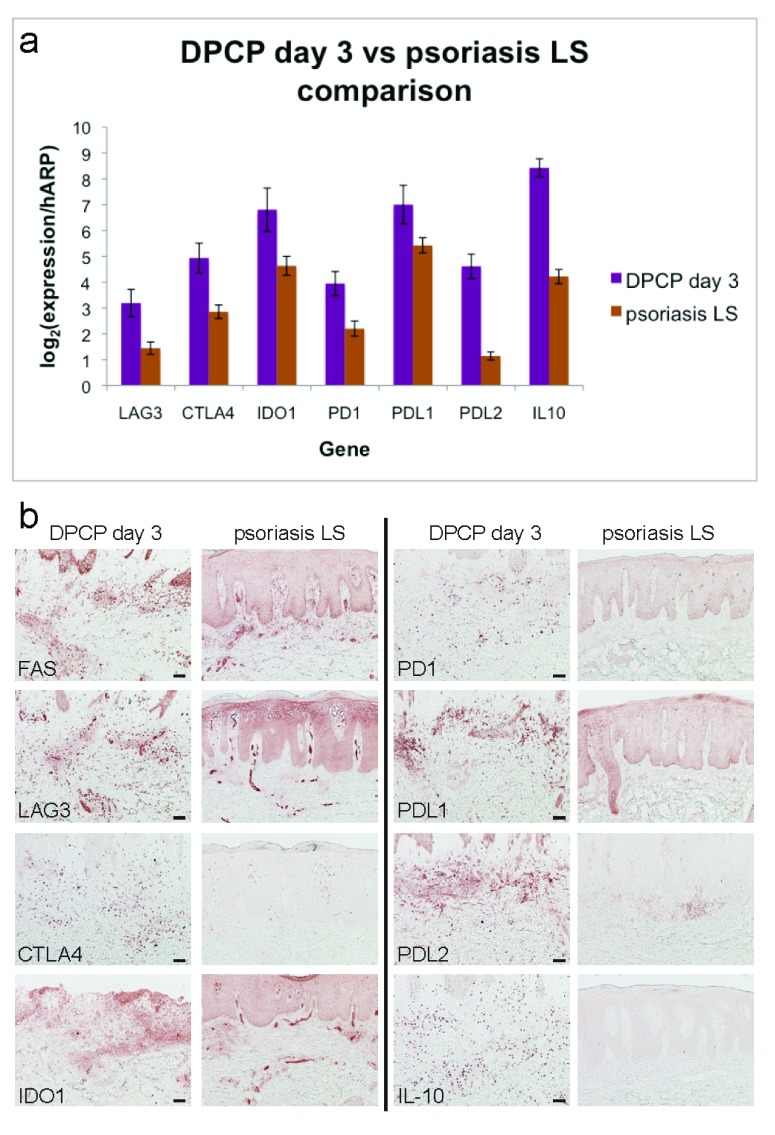
Psoriasis lesional skin has lower expression of various negative immune regulators than DPCP day 3 reactions by both qRT-PCR and immunohistochemical approaches. ( **a**) qRT-PCR analysis for negative regulators LAG3, CTLA4, IDO1, PD1, PDL1, PDL2, and IL-10. Shown are average normalized expression values for DPCP day 3 samples (n=11, purple bars) and psoriasis lesional skin (LS) samples (n=11, brown bars). All except PDL1 are *p*<0.05 by unpaired two-tailed *t*-test assuming equal variance. Error bars represent standard errors of the mean. ( **b**) Immunohistochemistry showing increased protein expression of negative regulators in DPCP day 3 samples compared to psoriasis LS. Shown are stains with antibodies specific to the indicated targets. Scale bar = 100 μm (applies to all images).

To confirm some of our microarray findings, we performed qRT-PCR and found that psoriasis lesional skin biopsies have significantly lower expression of many negative immune regulators compared to peak DPCP biopsies. These regulators include lymphocyte activation gene 3 (LAG3), cytotoxic T lymphocyte-associated 4 (CTLA4), indoleamine 2,3-dioxygenase (IDO1), programmed cell death 1 (PD1), programmed cell death 1 ligand 1 (PDL1), programmed cell death 1 ligand 2 (PDL2), and IL-10 ( Figure 2a). We confirmed the decreased expression of these and FAS (which by gene expression had 9.3- and 1.1-fold changes in the DPCP day 3 and psoriasis transcriptomes, respectively) at the protein level by immunohistochemistry ( Figure 2b).

## Discussion/conclusions

These data suggest that disease chronicity in psoriasis could be related to absence of several negative immune regulatory pathways, with the implication that strategies to obtain stable clearance/restore tolerance in skin lesions may need to focus on increasing these negative pathways. These negative immune mechanisms may be of more general importance for maintaining skin homeostasis as non-inflammatory in the presence of a large population of effector memory T cells that normally reside in skin ^10^. In addition, these negative immune regulators are likely involved in the therapeutic applications of DPCP, particularly alopecia areata where IL-10 has already been implicated ^4^. Further study of these regulatory mechanisms that are present in DPCP reactions, but not in psoriasis, could reveal novel factors in the pathogenesis of chronic inflammation. The DPCP system used here provides a tractable model for primary discovery of pathways potentially involved in immune regulation in peripheral tissues.
