# Supplementary material for: Psoriasis is characterized by deficient negative immune regulation compared to transient delayed-type hypersensitivity reactions
Source: F1000Res. 2015 Jun 11;4:149. [Version 1] doi: 10.12688/f1000research.6581.1 (PMC4505786; doi:10.12688/f1000research.6581.1)
Supplement: Peer review history

# Peer review: Referee response for version 1

**DOI:** 10.5256/f1000research.7066.r9716

**Published:** 2015-07-30

**Authors:** Luis Puig

**Affiliations:** 1 Department of Dermatology, Hospital de Sant Pau, Barcelona, Spain

The paper under review provides interesting research data with immunopathogenic implications for both delayed-type hypersensitivity (DTH) reactions and psoriasis. Using both microarray and quantitative RT-PCR the authors found that peak DTH reactions induced by diphencyprone (DPCP) 3 days post-challenge has significantly higher expression of negative immune regulators than psoriasis lesional skin. These regulators include interleukin-10 (IL-10), cytotoxic T lymphocyte-associated 4 (CTLA4), programmed cell death 1 (PD1), programmed cell death 1 ligand 1 (PDL1), programmed cell death 1 ligand 2 (PDL2), and indoleamine 2,3-dioxygenase (IDO1), and their increased expression was confirmed at protein level by immunohistochemistry. According to the authors, these findings might explain at least in part the disease chronicity in psoriasis and might provide new therapeutic avenues.

Of course, further studies are required to extricate the dynamics of immune activation in DTH vs psoriasis lesions; even data on DPCP reactions at 14 days can be found in another paper by the same authors ( Gulati *et al.*, 2014) detailed microarray and quantitative RT-PCR data on the time course of evolving lesions of psoriasis is lacking. Koebner phenomenon, guttate psoriasis and rebounds following clobetasol propionate suppression of plaque psoriasis might provide interesting models in this respect. The authors discuss the potential role of IL-10 regulation in DPCP immunotherapy of alopecia areata, and it might be interesting in this context to recall “Renbök phenomenon” or inverse Koebner phenomenon, namely the observation of normal hair growth in psoriatic plaques in patients with co-existing psoriasis and alopecia areata ( Mirmirani, 2015).

Pathogenic differences between DTH and psoriasis are not limited to the expression of genes involved in negative immune regulation, as illustrated by the lack of effect of blocking Th1/Th17 pathways with ustekinumab on the elicitation of ACD by patch testing ( Nosbaum *et al*., 2012), even though IL-1 β activation of dendritic epidermal T cells and production of IL-17 seems to be important in experimental contact hypersensitivity ( Nielsen *et al., *2014). On the other hand, nickel-sensitized psoriasis patients develop a delayed but otherwise typical allergic contact dermatitis (ACD) even in close proximity to pre-existing psoriasis plaques, with a predominantly Th17 response in psoriasis and a stronger Th2 and cytotoxic immune response in ACD ( Quaranta *et al*., 2014). Th9 cells are skin homing or skin resident cells which have been identified both in psoriasis and ACD, but IL-9 can have pro-inflammatory or regulatory activities depending on context ( Liu *et al*., 2014). The innate immune responses to damage associated molecular patterns and reactive oxygen species are of foremost importance for the elicitation of ACD *in vivo *( Martin, 2015).

Furthermore, the gene expression signatures and pathomechanisms of DTH reactions may differ according to the antigen ( Dhingra *et al.,* 2014) and duration of exposure. Nickel and other metal ions are able to elicitate ACD through the activation of toll-like receptor 4 (TLR4) ( Schmidt *et al*., 2010), direct activation of the NLRP·inflammasome with production of IL-1β ( Li *et al., *2014) and other mechanisms ( Vennegaard *et al., *2014).

Negative immune regulators include cytokines such as IL-10, IL-35 and transforming growth factor β (TGF β) and membrane proteins which are greatly interdependent and subject to cross-regulation, and several cell types, including Foxp3+ regulatory T cells, monocytes and B cells can be involved in down-regulation of inflammatory and autoimmune processes. Some of these cell types are characterized by their plasticity, so their phenotype might be subject to therapeutic modulation.

In conclusion, the paper by Gulati and coworkers represents a seminal work in the study of the comparative dynamics of immune activation and downregulation in several skin disorders, with potential therapeutic implications.

I have read this submission. I believe that I have an appropriate level of expertise to confirm that it is of an acceptable scientific standard.

# Peer review: Referee response for version 1

**DOI:** 10.5256/f1000research.7066.r9578

**Published:** 2015-07-20

**Authors:** Eugene William St. Clair

**Affiliations:** 1 Department of Medicine, Division of Rheumatology and Immunology, School of Medicine, Duke University, Durham, NC, USA

In this paper, Gulati and colleagues have tested the hypothesis that psoriatic skin lesions have significantly lower expression of negative immune regulators compared to peak DTH reactions from diphencyprone (DPCP). This study was done using two previously generated data sets described in previous publications from this group: 1) a study in 11 healthy human volunteers of recall responses to DPCP at 3-day (peak) and 14-day (resolution) using immunohistochemical techniques to visualize the infiltrating immune cells and gene expression (Affymetrix HGU133 Plus 2.0 Arrays) to perform a molecular profiling of the day 3 and day 14 DPCP responses, and in 6 cases 4-8 months later (reference 5); and 2) a meta-analysis derived (MAD) transcriptome of psoriatic skin from 3 different studies using Affymetrix HGU133 Plus 2.0 arrays in which microarray raw data were available through public databases (reference 6), termed the MAD-3 transcriptome.

The results show an imbalance in the expression of negative regulatory genes in DPCP day-3 peak reactions compared to psoriatic skin lesions, favoring the expression of more negative immune regulators in the former vs. the latter group (52 genes vs 7 genes). They generated odds ratios for the positive and negative immune regulators from a list of negative regulatory and positive regulatory genes derived from a GO search. The odds ratio for negative immune regulators was significantly higher in the DPCP day-3 (peak) group than the psoriatic skin group, while the odds ratios for the positive immune regulators were not statistically significantly different between the two groups. The enrichment for negative immune regulators held true also at day-14 (resolving) and day 120 (late). These findings were confirmed by qRT-PCR of several key genes and by immunohistochemistry.

This study is technically sound and the paper clearly written, with acceptable figures and tables. The title and the abstract are appropriate for the research described in the article. The methods are described in sufficient detail for the experiments to be replicated, although it would be useful to provide more detail about the methods underlying the calculations of the odds ratios. The interpretation of the results are reasonable based on the data and the conclusions provide a novel conceptual framework for understanding the fundamental mechanisms of skin homeostasis in psoriasis. 

I had one question about the results and their interpretation. In the study from reference 5, the results suggested different patterns of immune responses after DPCP challenge, referred to as subgroups A and B, where subgroup B had higher levels of negative immune regulators at day-3 than subgroup A. Interestingly, subgroup B fit the typical kinetics of a DTH response in which T cell and DC infiltrates peaked at day-3 and were diminished at day-14; whereas, subgroup A showed an unexpected increase in the numbers of T cells and DC’s at day-14 compared to day-3. It might be useful to know if the higher expression of negative immune regulators in DPCP skin compared with psoriatic skin was driven by the higher levels of these genes in subgroup B, or if it was generally true across both subgroups.

I have read this submission. I believe that I have an appropriate level of expertise to confirm that it is of an acceptable scientific standard.

# Peer review: Referee response for version 1

**DOI:** 10.5256/f1000research.7066.r9246

**Published:** 2015-07-16

**Authors:** Wilson Liao, Richard Ahn

**Affiliations:** 1 Department of Dermatology, University of California, San Francisco, San Francisco, CA, USA

In this welcome addition to the field of psoriasis transcriptomics, Gulati and colleagues sought to test whether expression of genes involved in immune regulation in transient condition, diphencyprone (DPCP) induced delayed-type hypersensitivity (DTH), was significantly different from expression of genes involved in immune regulation of a chronic T cell-mediated disease, psoriasis vulgaris. Employing microarray and qRT-PCR based approaches to profile the transcriptomes from biopsied samples, the authors found that negative immune regulatory genes were significantly overexpressed in the DPCP vs. healthy controls comparison, while such a pattern of overexpression was not observed in the psoriasis vs healthy controls comparison. This lack of overexpression of key negative regulatory genes was found in both the microarray and the qRT-PCR analyses. The authors conclude that the relative dearth of negative immune regulatory genes in psoriasis may be associated with the persistent inflammation found in psoriasis and that strategies to restore negative regulators may have therapeutic implications.

This paper is technically sound, with well accepted protocol employed in all wet experiments. The statistical analysis was also performed with well accepted and sufficiently rigorous methodology. The evidence supports the authors’ central claims. P-values for differential expression analysis were adjusted for multiple hypotheses by the Benjamini-Hochberg method to control for the FDR.

We believe that this paper will be of interest to researchers in the field of psoriasis transcriptomics and genetics, as well as the broader fields of autoimmunity and dermatology. The article could be strengthened by addressing the following comments and questions:

1. The authors note that “genes with low variation and low expression in most samples were filtered out prior to the analysis”. We think that the filtering threshold for variance and expression should be reported as well.
2. Odds ratios are reported in the results but it is not clear how they were estimated in the methods. Furthermore, interpretation of the odds ratios is unclear as well.
3. In Table 1, we have questions regarding the FCH and the p and FDR values. Is the FCH the absolute FC? Is it log2? We think that the labeling should be clearer. What do p and FDR values of “0E+00” indicate? Is it for p and FDR values that are high or low?
4. Could the authors’ results be confounded by the fact that psoriatic skin has a larger proportion of keratinocytes relative to DTH skin? If the negative regulators were primarily expressed in non-keratinocyte cell types, then biopsy of psoriatic skin compared to DTH skin might show a lower proportion of negative regulators based simply on cell proportions rather than intrinsic immunologic differences.
5. We feel that the biology of psoriasis and DTH (or contact dermatitis) can be expanded upon in the discussion section. For instance, a number of the genes listed in Table 1 are known T regulatory cell signature genes (including CTLA4 and IL2RA; please see Ferraro *et al*., 2014). Is the transcriptional profile of the observed DTH reaction consistent with the effects of a T regulatory cell effect, for example as described in Rosenblum *et al.* (2011)?
6. An apparent difference between the DTH model and psoriasis is that in the DTH model, antigen was transiently given, whereas in psoriasis it is possible that self-antigens are continually expressed. Might this contribute to the observed transcriptional differences? Have the authors’ examined the transcriptional profile of a DTH model in which antigen is chronically given?

We have read this submission. We believe that we have an appropriate level of expertise to confirm that it is of an acceptable scientific standard.

# Peer review: Referee response for version 1

**DOI:** 10.5256/f1000research.7066.r9426

**Published:** 2015-07-13

**Authors:** Jörg Prinz

**Affiliations:** 1 Department of Dermatology and Allergology, Ludwig-Maximilian University, Munich, Germany

Chronic immune-mediated inflammatory diseases represent major challenges in medicine. The reasons for the perpetuation of inflammation are only incompletely understood. Explanations for the chronicity of immune-mediated inflammatory diseases are essentially required to initiate causal therapeutic strategies.

The authors of the manuscript have chosen a novel approach to obtain insights into the molecular mechanisms associated with chronically perpetuating inflammation.

For this purpose, they focus on two clearly defined T-cell mediated disorders serving as model diseases for inflammation: delayed-type i.e. T-cell mediated hypersensitivity reaction (DTH) in response to an obligatory hapten antigen, diphencyprone (DPCP), and psoriasis vulgaris, a chronic T-cell mediated inflammatory skin disease. Both diseases are particularly suited for this approach. DPCP-specific DTH reactions are immune mediated, resolve naturally and have well-defined self-limited kinetics. This allows for the accurate definition of time points for the analysis of inflammatory peak reactions and resolving inflammatory activity. Psoriasis instead constitutes a persistent T-cell mediated skin inflammation.

The approach to obtain insights into the mechanisms perpetuating inflammation consists of the analysis and comparison of gene expression profiles in lesional and non-lesional skin probes of both diseases. Regarding psoriasis the authors refer to meta-analysis derived transcriptome data that cover the gene expression profiles from 193 lesional and non-lesional biopsy pairs as recently published. For DPCP-treated patients 11 lesional/non-lesional sample pairs were included for different time points during inflammation. Like the psoriasis transcriptome analyses the data acquisition in DCPC samples has been peer peer-reviewed in a high ranking scientific journal.

To achieve meaningful results regarding the posed question, the authors define two groups of genes for comparison. One comprehensive gene list includes positive regulatory or inflammatory genes that enhance inflammation, the other genes particularly related to negative regulatory or immunosuppressive functions. The composition of the gene lists is based on scientifically verified Gene ontology terms. In addition to the microarray data, for select genes expression levels are verified by quantitative RT-PCR and by immunohistochemistry analyses of protein expression in skin samples.

As a result the authors report, that at the time of the peak inflammatory response DPCP-induced DTH reactions contain significantly more genes related to suppression of inflammation than psoriasis lesions. This results in an altered balance between positive *vs*. negative regulatory transcripts in psoriasis compared to DTH reactions, which is maintained throughout DTH healing.

The authors conclude that in psoriasis, but also beyond this disease the lack of negative immune regulatory genes may be related to inflammatory disease chronicity. They furthermore propose that DPCP reactions may serve to examine regulatory immune pathways.

The study addresses a highly relevant question. The scientific approach is novel, original and represents a truly innovative solution to obtain novel insights into immune regulation. The comparison of psoriasis and DCPC reactions is reasonable because in both of them inflammatory reactions result from T-cell driven mechanisms. The methods are sound and appropriate for the analysis. The underlying data are extensive and due to the large sample size create a reliable and credible basis for the study. Accordingly, results and conclusions are well supported. The abstract provides an adequate summary of the study. The results are clearly presented. The discussion is short, draws a clear and valid conclusion and avoids unnecessary speculations. Indeed, the insights from the study provide a novel approach to understand the chronicity of inflammation.  

My personal assessment is that of an intriguing approach, which by itself is revealing and at the same time opens novel access paths into the investigation of chronic immune-mediated inflammation.

I support the publication of this article in the present form without any reservation. There are no changes necessary.

I have read this submission. I believe that I have an appropriate level of expertise to confirm that it is of an acceptable scientific standard.
